# Strategies to promote the dissemination of psychosocial digital health resources for those affected by cancer: scoping review

**DOI:** 10.1007/s00520-026-10657-3

**Published:** 2026-04-15

**Authors:** Isabel Ronan, Olinda Santin

**Affiliations:** 1 School of Computer Science and Information Technology, University College Cork, College Road, Cork, T12K8AF Ireland; 2 School of Nursing and Midwifery, Queen’s University Belfast, University Road, Belfast, Northern Ireland BT71NN UK

**Keywords:** Digital resource, Cancer, Dissemination, Oncology, Technology

## Abstract

**Purpose:**

The objective of this review is to map the evidence on dissemination strategies employed to promote awareness and uptake of psychosocial digital health resources for those affected by cancer while also addressing the barriers and facilitators to effective dissemination.

**Methods:**

A scoping review was undertaken using the Preferred Reporting Items for Systematic Reviews and Meta-Analyses Extension for Scoping Reviews (PRISMA-ScR) protocol. A wide range of keywords was used to form search terms for four databases, namely MEDical Literature Analysis and Retrieval System onLINE (MEDLINE), Cumulated Index in Nursing and Allied Health Literature (CINAHL), Embase, and PsycInfo.

**Results:**

We screened 5045 articles for inclusion after the initial automated removal of duplicate and ineligible items. A total of 4691 articles were excluded during title and abstract screening. A further 324 articles were excluded during the full-text screening stage. We included 30 articles in our analysis. Bibliometric analysis indicated that many low-to-middle-income countries are under-represented in the literature. Caregivers were seldom targeted in dissemination strategies. Other key themes emerging from our analysis include the need to close geographical, cultural, and digital divides and to increase the trust and credibility of interventions.

**Conclusion:**

This paper presents the first review consolidating research on psychosocial digital resource dissemination targeting those affected by cancer. These findings are of relevance to both oncology experts and researchers, who may use this review to reflect on how best to disseminate developed digital resources to cancer-affected populations. When considering the dissemination of an online resource in the future, researchers should focus on creating co-designed digital interventions for caregivers and targeting more diverse populations using both traditional and digital dissemination materials.

## Introduction

Cancer is currently one of the most common contributors to premature mortality in the world; the number of global cancer patients is expected to double over the next 50 years due to our ageing, growing population [1]. Cancer care also comes with rampant inequalities; there are different levels of treatment and resources available for those in various parts of the world [2, 3].

To combat the growing population affected by cancer, digital health solutions have been proposed to help support those providing and benefiting from oncology care [4, 5]. Digital health is the use of telecommunications or digital technology to improve healthcare [4]. In a world where cancer resources are becoming increasingly overstretched and under-resourced, a move towards digital interventions is necessary to meet the growing demand for cancer treatment and information, while also improving equitable access to care.

Within the digital resource landscape, psychosocial interventions are also becoming increasingly important. These interventions encompass a range of services and therapies to educate and console those living with a cancer diagnosis; such care can improve well-being and help in meeting the psychological, social, communication, and information-related needs of cancer patients, caregivers, and survivors [6].

Although the design of a psychosocial digital resource is very important in successful research, dissemination is often equally important. Dissemination is an active approach to the encouragement of intervention use via channels and planned strategies [7]. If a resource is not disseminated properly, then it will not reach the population it is designed to help. Digital resources have the potential to enable healthcare access and literacy; however, issues surrounding health technology adoption are common, with uptake in these new resources remaining low [8, 9]. Additionally, details on how to implement new interventions are often not adequately reported and indexed, leading to difficulties for both researchers and oncologists in deciding how best to disseminate key digital information to cancer-affected populations [10].

Research surrounding dissemination practices for psychosocial cancer resources is not well investigated; previous reviews have focused on trying to summarise such practices for general chronic diseases, rehabilitation, and clinician-level tools [9, 11, 12]. General dissemination strategies for patients and caregivers have been explored, along with the use of specific technology types [13–16]. Additionally, reviews have been conducted on digital health measures within specific locations [17, 18]. However, the dissemination of psychosocial digital cancer resources comes with its own set of disease-specific and care-specific challenges, which are not covered in the aforementioned reviews. To the best of the authors’ knowledge, our review is the first of its kind to focus exclusively on the subject of psychosocial digital resource dissemination for those affected by cancer.

We chose to undertake a scoping review due to the broad nature of the psychosocial cancer digital resource landscape. The purpose of this scoping review is to map the evidence on dissemination strategies employed to promote awareness and uptake of relevant digital health resources in cancer care and caregiving, while addressing the barriers and facilitators to effective dissemination among cancer-affected populations.

In this paper, we aim to consolidate past work, highlight dissemination challenges, and examine areas for future research. The research questions within this review were chosen to encompass the wide range of possible materials that could be considered within the cancer digital resource dissemination research landscape.

- What dissemination strategies have been used to promote cancer-related psychosocial digital health resources?
- What facilitators to dissemination are described in the literature?
- What barriers to dissemination are described in the literature?

This review’s main contribution to the literature is a contemporary collation of research regarding the dissemination of psychosocial digital resources for cancer patients, survivors, and caregivers. This review is of interest to any oncologists or researchers investigating how best to implement cancer-related psychosocial digital resources either in their own practice, in general medical settings, or within academia.

## Methods

This review was carried out from August to October 2025. Zotero and Microsoft Excel were used for reference management and data organisation, respectively [19, 20]. Searching and screening were undertaken by the first author, with subsequent study selection being conducted by both authors.

### Protocol and registration

We drafted our protocol using the PRISMA-ScR. We did not register our protocol.

### PICOC criteria

We used the Population, Intervention, Comparison, Outcome, and Context (PICOC) criteria to ensure our review remained focused on our chosen research questions. Our population was people affected by cancer; this population encompasses cancer patients, survivors, and caregivers. As outlined in Table 1, we performed a term breakdown to use in formulating search strings within our chosen databases based on the PICOC criteria.

**Table 1:** PICOC criteria—term breakdown

Criterion	Keywords	Synonyms
Population	Cancer	Carcinoma, leukaemia, lymphoma, malignancy, malignant, myeloma, neoplasm, oncology, sarcoma, tumor, tumour
	People affected by cancer	Care, care giver, caregiver, carer, family, partner, patient, relative, spouse, support person, supportive, survivor
Intervention	Dissemination strategy	Adoption, advertising, awareness, campaign, co-design, communication, strategy, diffusion, educational materials, engagement, implementation, knowledge mobilisation, knowledge translation, marketing, mass media, outreach, promotion, scale-up, spread, uptake
	Digital health	Digital health, SMS, app, application, chatbot, decision aid, digital intervention, digital program, digital resource, digital tool, eHealth, internet-based, mHealth, online intervention, online program, online resource, online support group, online tool, platform, portal, telehealth, telemedicine, text message, website
Comparison	Not applicable	Not applicable
Outcomes	Uptake	CTR, acceptability, acceptance, adoption, appropriateness, awareness, click-through rate, digital divide, download, engagement, equity, feasibility, fidelity, health literacy, impression, install, penetration, reach, retention, satisfaction, share, sustainability, usability, utilisation, view, click-through rate
Context	Cancer-related digital health resources	Oncology care, cancer care continuum, screening, diagnosis, treatment, survivorship, palliative care, end-of-life care, supportive oncology

### Eligibility criteria

Eligibility criteria were defined to filter articles during the title and abstract screening stage. Materials within the last 20 years (i.e. January 2005 to August 2025) were chosen in order to track trends and changes in the digital resource landscape over a substantial period of time. Articles were limited to those in English, as it was not practical to hire a translator to interpret research in other languages. Only peer-reviewed academic articles from journals and conferences were included to ensure high-quality review results; excluding other sources, such as grey literature, ensures the studies discussed in this review have been subject to rigorous peer review. Other types of materials were not considered due to difficulties in proving validity from low-quality sources. Additionally, other literature reviews were excluded from our analysis as they are secondary sources of information.

We required that all materials chosen for review were either open access or were subject to some university-provided access agreement, allowing us to view the full text without further payment. We excluded materials that required purchase for financial reasons. Finally, the articles under appraisal had to be relevant to at least one of our research questions to be considered for inclusion. Resources targeting healthcare professionals were not considered relevant. All possible digital resources were considered relevant. Additionally, studies had to include some form of dissemination strategy and measurement of resource uptake.

### Information sources

Digital libraries were selected for searching based on their relevance to our research questions and their use in past literature [13, 21, 22]. We chose the CINAHL, Embase, MEDLINE, and PsycInfo libraries [23–26]. Databases were all searched at the end of August. Exported studies were refined over the September period.

### Search

**Fig. 1:**
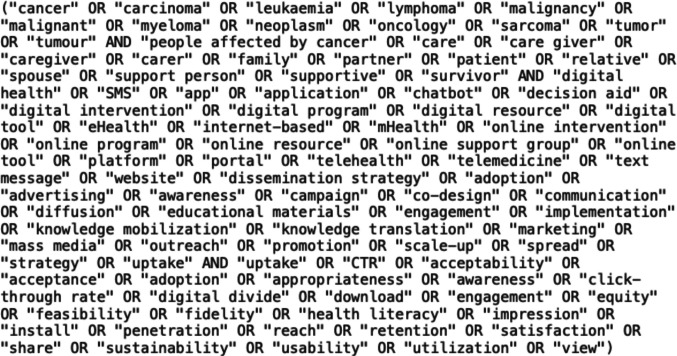
Base search string

Each library has its own search engine features, facilitating advanced search strings and keyword usage. The keywords for search queries used were derived from the PICOC term breakdown process outlined in Table 1. Figure 1 shows the base search string used before database-specific modification, with a series of logical operators used to distinguish between each of the PICOC criteria.

### Selection of sources of evidence

After title and abstract screening, the full text of each article was assessed to determine review relevance. In order to be considered relevant, studies had to be related to people affected by cancer; studies were considered irrelevant if they only targeted healthcare staff or policy makers, with no inclusion of caregivers, survivors, or patients. Oncology clinicians were included only if research was focused on encouraging them to share resources with patients, survivors, and carers, or if they were included with the aforementioned people as targets for the digital resource. Studies were included if they clearly described details of any dissemination strategy to raise digital health resource awareness; studies that did not report any usage numbers or uptake measures whatsoever were excluded. Articles describing the dissemination of non-digital interventions were also considered irrelevant, such as surveys, exercise programmes, or cancer screening programmes. 

The first author conducted preliminary full-text screening; material choices were reviewed by the second author and sent back to the first author for appraisal. Relevance was reconsidered following first-round full-text screening, leading to the need to further refine what type of digital resources were to be considered eligible for inclusion. Subsequently, materials were excluded if they were not general psychosocial interventions; psychosocial digital resources had to include some aspect of generalisability in their design to be considered eligible for review, making them applicable to broader cancer-related populations, not just those they were designed to be used for. Full-text screening was subsequently refined in a cyclical process, passing from first to second author repeatedly until both authors were satisfied with the papers included and all disputes regarding materials eligible for inclusion were resolved.

### Data charting process and data items

Data charting was undertaken using Microsoft Excel. A pilot charting process was undertaken with five articles to ensure all key elements of the study were captured. A free-text process was followed during initial charting stages to capture all key themes. Subsequently, once all articles were charted, a more rigorous thematic and itemised categorisation was undertaken. These key data items and themes are reported on in the “Results” and “Discussion” sections, respectively.

The number of people who used the digital resources within each study was collected, along with bibliometric data, such as research country and publication information. Information surrounding facilitators and barriers was collected and subsequently refined into distinct items and thematic categories.

### Synthesis of results

Data was processed using the Python programming language to allow for the easy and flexible visualisation of results. Jupyter Notebooks were used to import data, count the number of reoccurrences of particular variables in the analysis, and display results in counter objects or graphs. Additionally, this code usage ensured that subsequent theme refinement was easily undertaken by rerunning analysis files. Various data points from the data charting stage outlined in “Data charting process and data items” were synthesised using this coding process.

## Results

### Selection of sources of evidence

**Fig. 2:**
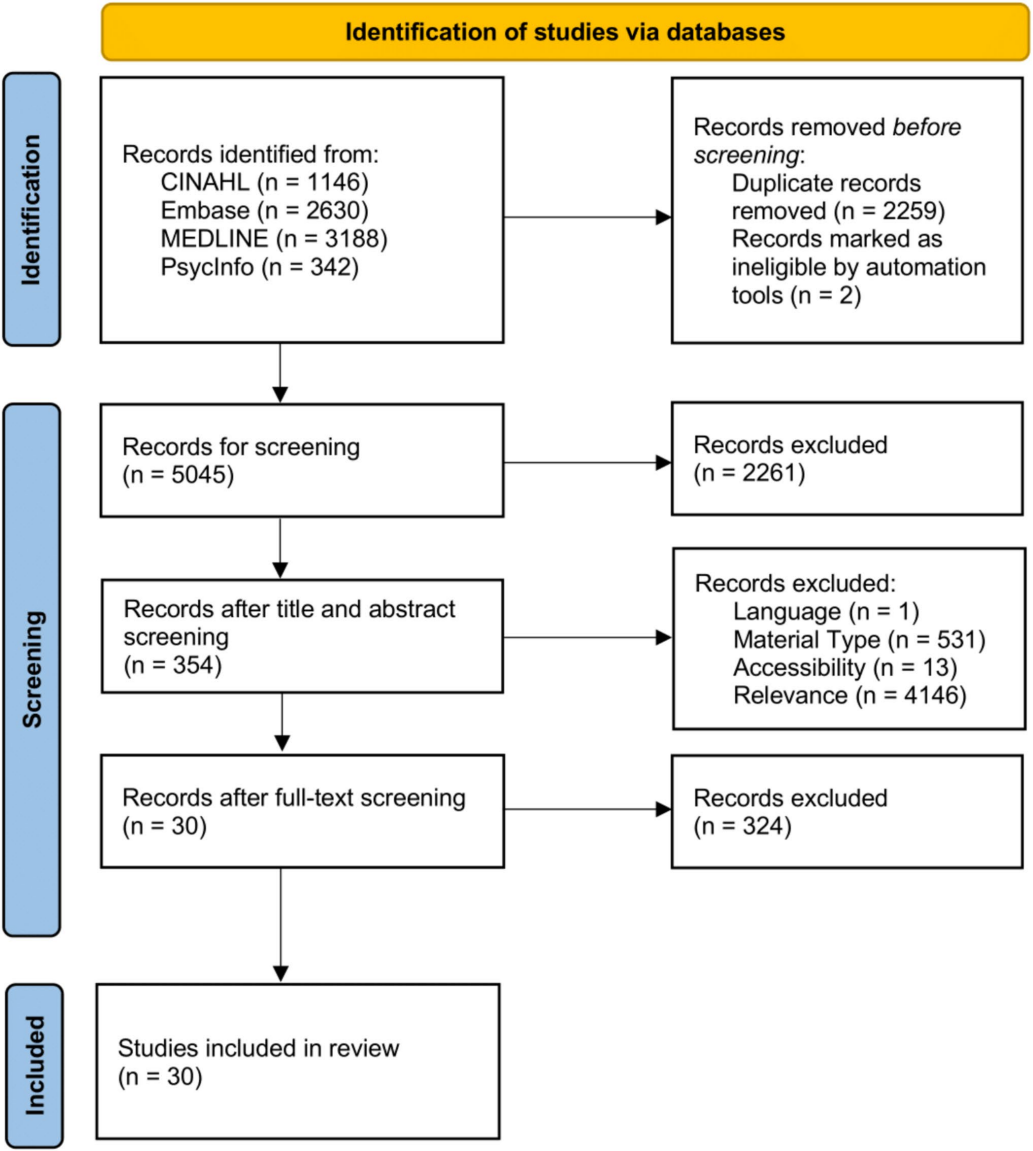
Article selection process

The search process, along with its associated results, is outlined in a PRISMA-ScR-style flowchart in Fig. 2. Two articles were marked as ineligible when database results were imported into Zotero; one article was a retracted article, and another item was blank upon import, with no identifying information. After all screening stages were completed, 30 articles remained for review. A range of qualitative, quantitative, and mixed methods studies is included in this review. Due to the diverse range of study design types present in the literature, we did not conduct an assessment of the quality of included studies.

### Characteristics of sources of evidence

#### Research country

Most studies came from Western regions, with North America (*n* = 18), Australia (*n* = 7), and Europe (*n* = 6) featuring most often in the literature. Kenya (*n* = 1) and Saudi Arabia (*n* = 1) are the only two countries present in the reviewed materials that fall outside the Western scope.

#### Recruited population numbers

The majority of studies that recorded target population numbers only managed to recruit small numbers of people. The mean number of people initially exposed to the digital interventions was approximately 308. The mean number of people retained throughout the duration of the intervention was approximately 157. The minimum number of people involved in any study was 24 initially, and 10 upon intervention completion. The maximum number of people involved initially and upon intervention completion was 2961 and 1070, respectively. Studies that recorded website hits and various other metrics (*n* = 3) are not included in these numbers [27–29].

### Results of individual sources of evidence

Table 2 outlines all relevant outcomes data for each source of evidence.

**Table 2:** Data extraction table

Paper	Year	Country	Target group	Cancer type	Type of resource	Purpose	Dissemination level	Dissemination strategy
[27]	2010	USA	Patients	Prostate; colorectal	Educational Video(s)	Self-management	Local	Direct message; word of mouth
[28]	2017	USA	Survivors	Prostate	Website	Education	Regional	Direct message; traditional media
[29]	2022	USA	Patients	Any	Educational Video(s)	Self-management	Regional	Digital media; traditional media
[30]	2020	USA	Survivors	Any	Website	Mental health	National	Direct message; digital media; word of mouth
[31]	2020	USA	Patients; caregivers	Breast	App	Mental health	Local	Traditional media; word of mouth
[32]	2020	USA	Patients	Breast	App	Mental health	Local	Traditional media; word of mouth
[33]	2020	USA	Survivors	Breast	Website	Self-management	Local	Direct message; traditional media
[34]	2020	UK	Survivors	Any	Online program	Self-management	National	Digital media
[35]	2020	UK	Patients; survivors	Breast	N/A	Self-management	National	Digital media; traditional media; word of mouth
[36]	2020	Australia	Patients	Breast; colorectal	App	Communication	Local	Word of mouth
[37]	2022	Kenya	Patients	Breast	Website	Education	National	Traditional media; digital media; word of mouth
[38]	2024	USA	Patients	Prostate	App	Self-management	Local	Word of Mouth
[39]	2024	USA	Patients; caregivers	Any	App	Self-management	Regional	Direct message
[40]	2024	USA	Patients	Any	Telehealth platform	Mental health	Local	Direct message; word of mouth
[41]	2022	Germany	Caregivers	Any	Online program	Mental health	National	Digital media; traditional media; word of mouth
[42]	2024	Australia	Survivors	Breast	Online program	Mental health	National	Direct message; digital media; word of mouth
[43]	2022	USA	Survivors	Any	Online program	Self-management	National	Direct message; digital media; traditional media
[44]	2025	USA	Survivors	Ovarian	Website	Education	Regional	Direct message
[45]	2018	Australia	Survivors	Any	Online program	Mental health	National	Direct message; digital media
[46]	2025	Australia	Patients; survivors	Breast	Online program	Mental health	National	Digital media
[47]	2023	USA	Patients	Breast	Telehealth platform	Self-management	Local	Direct message; word of mouth; traditional Media
[48]	2021	Saudi Arabia	Patients	Any	Portal	Education; self-management	Local	Word of mouth
[49]	2019	USA	Patients; caregivers	Breast	Portal	Communication	Local	Direct message; word of mouth
[50]	2016	Germany	Patients	Colorectal; prostate; breast	Website	Education	National	Traditional media; word of mouth
[51]	2014	Australia	Patients	Prostate	Online platform	Mental health	National	Traditional media; digital media; word of mouth
[52]	2011	USA	Patients	Head; neck	Telehealth platform	Education	Local	Traditional media; word of mouth
[53]	2025	Australia	Patients; survivors	Breast; prostate; colorectal	Online program	Mental health	National	Digital media; word of mouth
[54]	2023	UK	Patients	Any	App	Self-management	Regional	Digital media
[55]	2021	Australia; UK; Canada; USA	Patients; survivors	Any	App	Self-management	International	Digital media
[56]	2010	Canada	Patients	Breast	Portal	Self-management; mental health	Local	Word of mouth

### Synthesis of results

#### Target population and cancer types

The majority of the studies targeted cancer patients (*n* = 21). Cancer survivors (*n* = 12) and cancer caregivers (*n* = 4) also featured as target populations in the literature. Of the studies that focused on patients, 13 studies targeted breast cancer-related populations [31–33, 35–37, 42, 46, 47, 49, 50, 53, 56]. Many studies included mixed cancer populations (*n* = 11) [29, 30, 34, 39–41, 43, 45, 48, 54, 55]. Studies including prostate (*n* = 6), colorectal (*n* = 4), ovarian (*n* = 1), head (*n* = 1), and neck (*n* = 1) cancers also feature in the literature [27, 28, 36, 38, 44, 50–53].

#### Intervention details

The majority of studies focused on online programmes (*n* = 8), apps (*n* = 7), or websites (*n* = 6) [28, 30–34, 36–39, 41–46, 50, 51, 53–55]. Other interventions, such as portals (*n* = 3) and telehealth platforms (*n* = 3), were less frequently described in the literature [40, 47–49, 52, 56]. Online programmes could further be decomposed into website or app resources; however, they are often multimodal and can encompass a range of both technological and in-person supports.

The majority of interventions focusing on cancer patients alone made use of apps (*n* = 4). Overall, the types of intervention focusing on cancer patients were the most diverse, with educational videos (*n* = 2), online programmes (*n* = 1), patient portals (*n* = 2), telehealth platforms (*n* = 3), and websites (*n* = 2) also featured. Interventions focused solely on cancer survivors made use of online programmes (*n* = 4) and websites (*n* = 4). There was only one online programme exclusively targeting caregivers. Other mixed-group studies used apps (*n* = 3), online programmes (*n* = 2), and patient portals (*n* = 1), if specified.

The main purpose of the majority of interventions was to provide some form of self-management tool (*n* = 13); self-management in this context extends to both symptom reporting and personal decision-making [27, 29, 33–35, 38, 39, 43, 47, 48, 54–56]. Mental health aids (*n* = 11), educational resources (*n* = 6), and communication tools (*n* = 2) also featured in the reviewed papers [28, 30–32, 36, 37, 40–42, 44–46, 48–53, 56].

#### Co-design and dissemination

**Fig. 3:**
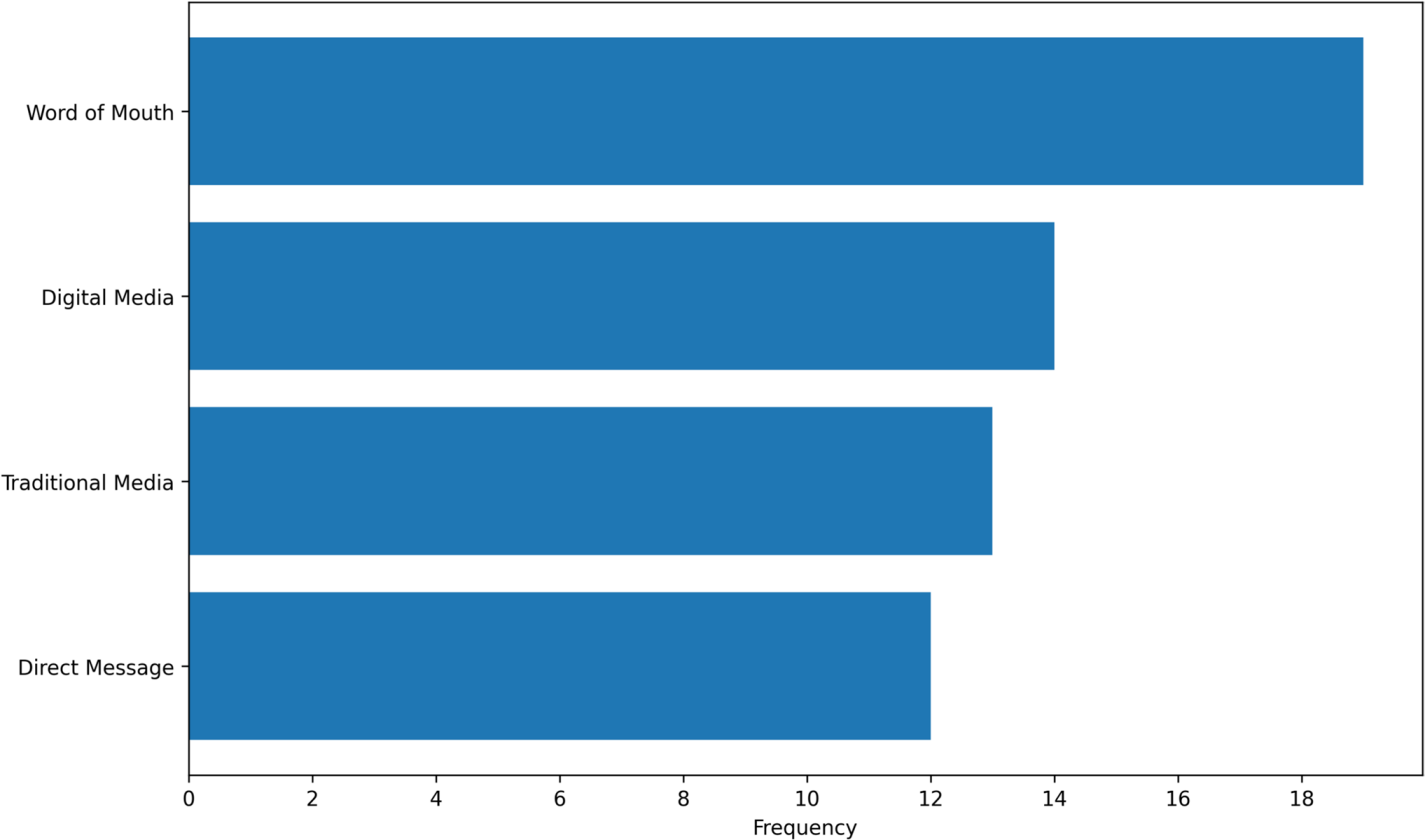
Dissemination channels

Only five studies explicitly described or outlined their use of co-design in the creation, implementation, or dissemination of their digital resource [28, 29, 33, 37, 53]. As outlined in Fig. 3, the majority of studies included some form of word-of-mouth dissemination strategy (*n* = 19) [27, 30–32, 35–38, 40–42, 47–53, 56]. Word-of-mouth strategies usually feature face-to-face clinician contact or endorsement from a cancer organisation representative. One out of the 13 studies did not report the involvement of some form of cancer organisation or clinic in their dissemination strategy [55]. Digital media (*n* = 14), such as social media and recruitment or organisational websites, were also used frequently in the literature [29, 30, 34, 35, 37, 41–43, 45, 46, 51, 53–55]. Direct message (*n* = 12), such as text, email, mail, or phone call, and traditional media (*n* = 13) usage, such as flyers, posters, pamphlets, newspapers, or brochures, were also used [27–33, 35, 37, 39–45, 47, 49–52]. Two studies provided examples of their materials in their publication; the majority of studies (*n* = 28) did not show examples of their dissemination materials [29, 55].

**Fig. 4:**
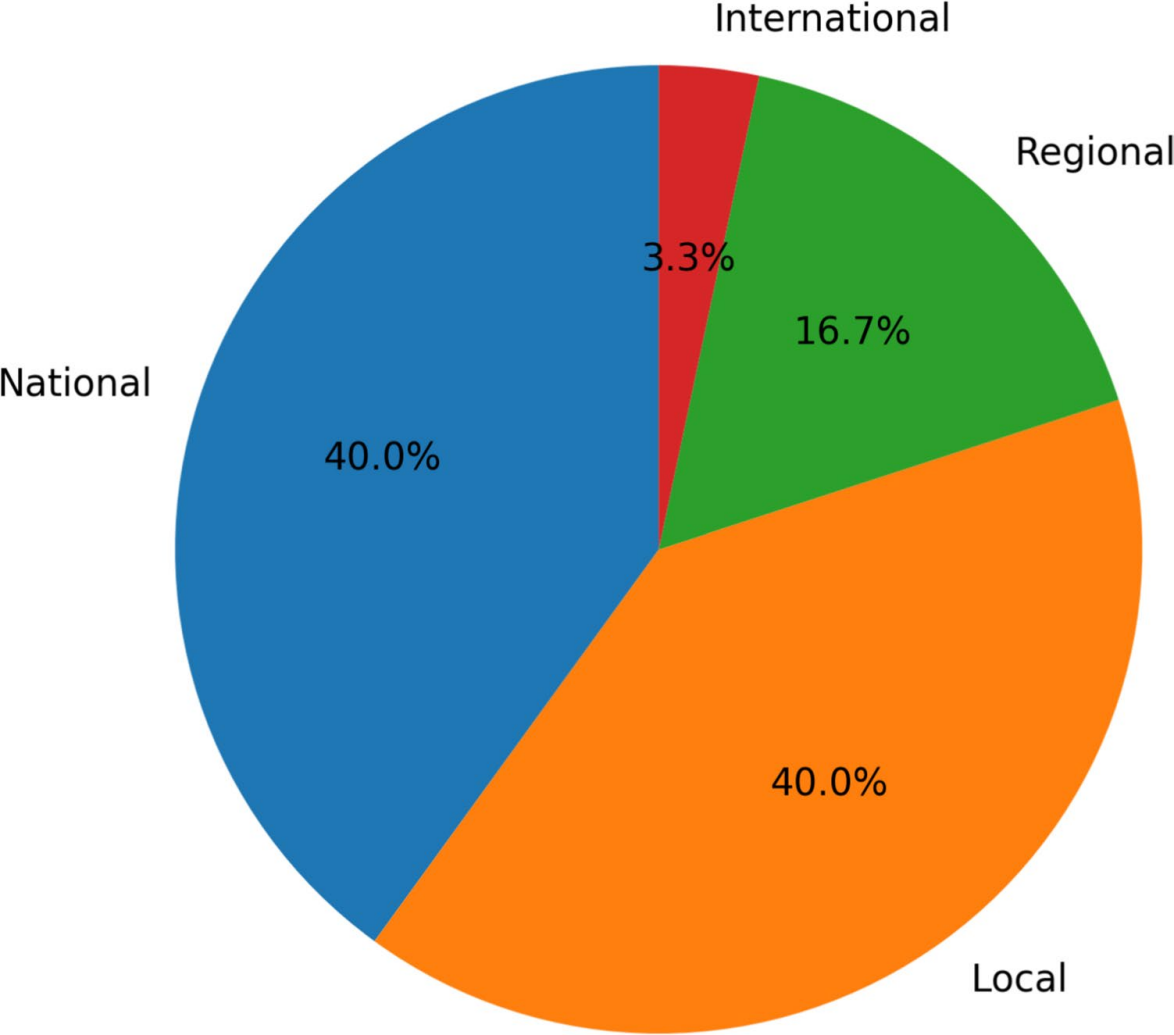
Level of dissemination

As outlined in Fig. 4, the majority of studies involved either a national (*n* = 12) or local (*n* = 12) dissemination strategy [27, 30–38, 40–43, 45–53, 56]. This figure shows the range of dissemination levels considered in the recruitment of people; the distinction made between regional and local strategies refers to whether a study involved either multiple sites or locations within the same county or province or simply involved a single site or locality. Eighteen studies highlighted the need for more diverse or larger target populations [28–30, 34–36, 38–40, 42–44, 46, 48, 51, 53–55]. Additionally, 25 studies reported that sample diversity or size was a limitation of their work [29–32, 34–39, 41–44, 46–56].

#### Facilitators

Clinician, organisation, or researcher promotion and troubleshooting (*n* = 12) frequently facilitated digital intervention usage [28, 31, 32, 36, 37, 41, 47, 52–54, 56]. People were inclined to use digital resources when recommended by trusted medical or cancer-related figures in their lives. Additionally, the duration, relevance, and structure of interventions seemed to be cited as frequent reasons for participation (*n* = 9) [27, 29, 30, 32, 35, 38, 44, 46, 51]. Interventions were used by people who felt that there was direct relevance, such as websites for an exact form of cancer or treatment, as opposed to interventions about cancer in general [30, 44]. Apps (*n* = 2), educational videos (*n *= 2), and online programmes (*n* = 2), which are flexible, both in structural and temporal terms, were appealing to people. Apps (*n* = 2) allowed relevant persons to access interventions at times that suited them and in portable formats [38, 57]. Educational videos (*n* = 2) were in an easily digestible format [27, 29]. One video-related study did not provide information on the duration of the content [27]. The other study used 20 videos of approximately 1 min in length [29]. Online programmes (*n* = 2) made mental health supports available to people from their own homes, again allowing for temporal and structural flexibility [46, 51].

In four studies, people indicated that social opportunities attracted them to digital resources [34, 35, 45, 46]. Reminder emails, texts, notifications, or calls also encouraged people in six studies to use a digital resource [30, 43, 45, 47, 52, 53]. Three studies mentioned curiosity as a reason for using the digital resource [40, 48, 51]. Four studies did not include any significant discussion of facilitators [42, 49, 50, 55].

#### Barriers

The most frequently discussed barriers to digital intervention usage included target population issues (*n* = 22) and technological issues (*n *= 20); only two studies did not mention either of these barriers [34, 45]. Target population issues included language barriers, lack of awareness about the intervention type, purpose, or benefit, confusion surrounding the intervention, emotional or information overload, lack of trust in the intervention, illness-related issues, and stigma surrounding cancer or the type of intervention [27–33, 36, 38–42, 46–54]. Technological issues included confidence in the intervention, skill level, and accessibility [29, 30, 32, 35–38, 40–44, 46–48, 50, 52, 54–56]. Digital literacy was highlighted in three studies as a significant barrier to the use of resources by appropriately diverse populations [32, 34, 39].

Time issues (*n* = 11), such as the lack of free time or the lengthy nature of a resource, also frequently appeared as barriers in the literature [29, 31, 32, 34, 40, 41, 46, 47, 49, 52, 53]. To a lesser extent, clinical issues (*n* = 5), such as disruption to the patient-provider workflow or relationship, were also mentioned [32, 34, 41, 45, 47].

## Discussion

### Principal findings

#### Overview

To our knowledge, this review is the first to synthesise psychosocial cancer digital resource dissemination strategies. This review includes 30 articles relevant to the dissemination of digital interventions. From this literature, key insights can be derived surrounding cultural, geographical, and digital divides, trust and credibility, and the target population focus.

#### Cultural, geographical, and digital divides

Overall, the papers featured in this review focus predominantly on Western countries; the lack of geographical and cultural diversity reflects the rather narrow range of perspectives found within the literature. This focus leads to academic insights that may not be applicable outside of the Global North. The need for diverse population samples is mentioned in many studies in order to create more robust findings; even within Western settings, there is a tendency to target specific ethnic or gender-based groups, leading to results reporting on homogenous populations.

Western ideological belief in individual control leads to certain forms of dissemination strategy becoming more successful in the locations for which they are designed; however, these dissemination strategies do not take different cultural perspectives about cancer into account, leading to the ethnocentric creation of dissemination materials for predominantly white, Western audiences [58]. Many low-to-middle-income countries lack the resources found in higher-income countries, yet have over-represented patient populations with advanced cancer; tailored approaches to care are necessary in these countries that take cultural and religious differences into account [59].

Furthermore, there exists a digital divide between those who are technology-savvy and those who are not, leading to growing societal access challenges [60]. Many studies in this review highlight that their findings are limited to a digitally literate population or that future work should focus on targeting a diverse range of digital literacy levels. As outlined in the literature, those who are not technologically confident may not be interested in accessing digital resources due to their own perceived inadequacies in relation to technology [38, 44]. However, this circumstance only leads to a wider gap between those who benefit from technology and those who do not; the digital divide in itself becomes a manifestation of socio-demographic and socio-economic disparities that exist within populations around the globe [60].

Future research should help to target those less technologically literate, along with people from under-represented areas, in order to improve the technological diversity of relevant populations. Additionally, both traditional and digital media should be designed to encourage those who are not technologically confident to become involved in the development of digital resources.

#### Trust and credibility

Co-design, within the context of our review, is the process of engaging patients, healthcare professionals, and other stakeholders to develop digital resources [61]. Very few studies within our chosen literature report the use of co-design in their research; however, many studies used some form of clinician or organisation-based recruitment strategy to disseminate their digital resource. From our review, it seems that when clinicians or organisations are involved in the dissemination of resources to target populations, trust in and thereby usage of the intervention grows. This encouraging finding highlights that involving these key figures, not only in the dissemination but also in the development of resources, may further enhance resource uptake. Potential target groups may be more inclined to interact with proposed interventions when they know that trusted figures in their lives, such as healthcare professionals, cancer survivors, or organisational representatives, were involved in the design process.

There is a juxtaposition between the lack of co-design usage in the literature and the use of stakeholders in the dissemination of resources; researchers do not make it clear whether collaborative partners should feel a sense of ownership over the resources they are disseminating. If key stakeholders are involved in the design of such resources, there may be increased trust in this research, leading to dissemination strategy success. Future research should include key stakeholders in not only dissemination processes but in design processes too, potentially increasing digital resource uptake.

#### Patient versus caregiver focus

Very few of the studies selected for this review focused on cancer caregivers. Cancer caregivers are becoming increasingly important as more and more cancer care moves to outpatient delivery settings with home monitoring [62]. The majority of digital resources are not created with these key people in mind; carers’ understanding of health information is crucial to appropriately support patients and survivors in cancer-related settings [63].

From a dissemination perspective, there are unique challenges associated with providing information to caregivers. Busy caregivers are quite often short on time; they are greatly affected by the so-called time toxicity of cancer [57]. Additionally, caregivers can have unmet needs surrounding mental health and well-being, which can be exacerbated by caring for loved ones with cancer [64]. Due to the lack of information found in the literature related to the dissemination of caregiver digital resources, more robust research is needed to provide deeper insights into this important group. Future research should focus on the creation and dissemination of resources to support cancer caregivers.

### Limitations

In this review, we chose four databases; by limiting ourselves to these databases, we could potentially exclude publications from other reputable sources. Additionally, while both authors were involved in the article selection process, the first author conducted preliminary title and abstract screening alone; this increases the likelihood of selection bias. While the use of the PRISMA-ScR protocol limits some potential for bias, it does not eliminate it entirely. Future reviews on this subject could include more researchers in the article selection process.

Our eligibility criteria limited our findings to English-language articles. While we report in this study a lack of materials from certain geographical locations, we cannot say that these materials do not exist; they may simply be in other languages. Additionally, we excluded inaccessible materials due to financial constraints; as a result, we may have excluded important studies in the digital resource field. Furthermore, only conference and journal articles were considered, ensuring high-quality papers were analysed. However, we potentially limited the breadth of review results to a narrow range of material types. Prospective reviews with similar research questions should include less stringent eligibility criteria to gain a deeper understanding of the cancer-related digital resource field.

## Conclusion

This PRISMA-ScR scoping review was undertaken to investigate the current stage of research on dissemination strategies for digital resources aimed at people affected by cancer. Our findings are based on 30 research articles. Bibliometric analysis revealed that the reviewed literature was predominantly focused on Western nations, revealing cultural homogeneity in our findings. Additionally, we found that access to technology is deeply connected to geographical and cultural disparities in the literature. We also found that dissemination was hindered by issues surrounding trust, credibility, and the participation of key stakeholders in resource design and dissemination. Finally, research undertaken into the dissemination of digital resources for caregivers, along with standardised recruitment measures, is desperately needed in this field.

The main contribution of this study is a review of psychosocial digital resource dissemination strategies targeting people affected by cancer. By addressing the issues highlighted in this review, it will be possible to create a more equitable, effective, and informed cancer care landscape for future populations.

## Data Availability

All data, materials, and code relevant to this submission can be found at 10.5281/zenodo.19456035.
